# Return to sport in athletes after surgical ankle fractures: A systematic review

**DOI:** 10.1002/jeo2.70392

**Published:** 2025-10-27

**Authors:** Giovan Giuseppe Mazzella, Guido Bocchino, Andrea De Fazio, Giacomo Capece, Fabrizio Forconi, Giulio Maccauro, Raffaele Vitiello

**Affiliations:** ^1^ Department of Orthopedics and Geriatric Sciences Catholic University of the Sacred Heart Rome Italy; ^2^ Department of Orthopedics, Ageing and Rheumatological Sciences Fondazione Policlinico Universitario A. Gemelli IRCCS Rome Italy; ^3^ Villa Stuart Sport Clinic, FIFA Medical Center of Excellence Rome Italy

**Keywords:** ankle, fractures, ORIF, rehabilitation, return to sport

## Abstract

**Purpose:**

Ankle fractures represent a significant health concern for athletes, comprising 15%–25% of all sports‐related injuries. With increasing sports participation across all age groups, the public health impact of these injuries is expected to grow. Open reduction and internal fixation (ORIF) is a common approach to managing ankle fractures, particularly for athletes requiring rapid return to sports. However current information on the return to sports post‐injury is largely anecdotal.

**Methods:**

A comprehensive literature search was conducted using keywords related to ankle fractures and sports return. Databases searched included Medline (PubMED), Cochrane and Google Scholar up to 12 April 2024. Articles published in English, Spanish, French, Portuguese, and Italian were considered. A total of 17 studies involving 1422 patients were analysed. Inclusion criteria covered randomised controlled trials and case series of over 10 patients focused on athletes with closed ankle fractures. Studies on paediatric fractures, soft tissue injuries, and non‐sports‐related fractures were excluded.

**Results:**

The review analysed various types of ankle fractures, with an average patient age of 31.4 years. ORIF was the predominant treatment (89.4%), and the average return‐to‐sport rate was 87.1%. Time to return varied, with an average of 134.5 days. Recovery time was longer for more severe fractures, such as bimalleolar and trimalleolar fractures, which showed times of up to 720 days. Functional outcomes improved with early rehabilitation, and the average Foot and Ankle Outcome Score (FAOS) for sports was 85.6 after 12 months.

**Conclusion:**

Returning to sports after an ankle fracture is a critical concern for athletes. This systematic review provides valuable insights into the prognosis and timeline for resuming sports activities. By synthesising current evidence, it offers guidance for clinicians and athletes on managing recovery and optimising return to sports. Standardised, high‐quality studies are needed to provide clearer guidelines.

**Level of Evidence:**

Level III.

**For Clinical Trials:**

PROSPERO registration number 594839.

**Abbreviations**
AORIF: arthroscopic‐assisted open reduction and internal fixation
ARIF: arthroscopic reduction and internal fixation
CRIF: closed reduction and internal fixation
ORIF: open reduction and internal fixation
RTS: return to sports

## INTRODUCTION

Ankle fractures secondary to sports injuries represent a significant health concern for athletes accounting for 15%–25% of all sports‐related injuries [12, 13]. With the increasing participation in sports across all age groups, the public health impact of these fractures is expected to grow [47]. Effective management of ankle fractures, including open reduction and internal fixation (ORIF), is crucial for athletes who require a swift return to sports activities [10]. Athletes can be severely debilitated and functional recovery and return to pre‐injury levels of sports activity can be severely compromised [9]. An athlete is often defined based on participation in organised sports with the primary goal of excelling in performance, typically requiring systematic and intense training [2, 44]. In contrast, exercisersengage in physical activity primarily to enhance health, fitness, or physique rather than for competitive achievements [25]. Athletes can be further classified by their level of competition and training volume. Elite athletes, such as Olympians or professionals, often train over 10 h per week with the goal of achieving excellence in high‐level competitions [26]. Competitive athletes, including high school or college participants, train at least 6 h per week for official events, while recreational athletes train more casually, typically exceeding 4 h per week for enjoyment or informal games [26]. Training volume (hours/week) and competition levels are useful but imperfect metrics for distinguishing athletes, as both athletes and exercisers may engage in high‐intensity physical activity. Depending on the type of fracture, the stability provided by ORIF can allow for early rehabilitation and a return to sport within 2–4 months. However, ORIF is associated with substantial surgical exposure and inherent complications. Ankle arthroscopy is indicated for both diagnosis and treatment of a large spectrum of common ankle disorders, such as osteochondral lesions, ankle instability, loose body removal, chondral or syndesmosis injury, acute trauma and sequelae [7]. Closed reduction and internal fixation (CRIF) or arthroscopic reduction and internal fixation (ARIF) and arthroscopic‐assisted open reduction and internal fixation (AORIF) have been introduced to provide fracture stability and treat associated intra‐articular injuries, minimising surgical exposure and further optimising outcomes and return to sport [45]. The definition of return to sport (RTS) can vary widely. Some studies consider RTS as the athlete returning to full competition or game play, while others include the resumption of practice or training, often with specific goals or competition levels in mind. This lack of consistency makes it difficult to accurately assess outcomes after sports‐related surgeries. By using clear terms that describe different stages of recovery, like ‘return to participation’ and ‘return to performance’, we can better understand the athlete's progress. For example, return to participation refers to the stage where an athlete is active in rehabilitation or training, but not yet ready for full competition. They might be physically active, but not fully prepared, whether medically, physically, or psychologically, to compete at their desired level. RTS marks the point where the athlete is back to their sport, though they might not yet be performing at their best or pre‐injury level. For some, just reaching this stage can be seen as a success. Finally, return to performance means the athlete has fully regained or surpassed their pre‐injury form and is performing at or above their previous level. This stage may even represent personal bests or expected growth in performance. By distinguishing these stages, we gain a clearer picture of the recovery process and the athlete's readiness to compete. Furthermore, in our review, we focused solely on RTS, as it was the most frequently mentioned parameter in the literature. This focus allowed us to concentrate on the most widely used outcome measure while recognising the importance of clarifying the various stages of return to athletic activity [8]. Information regarding the return to sports activities after such surgical interventions is often anecdotal and lacks a robust evidence base. This is a relevant problem, as a clear understanding of postoperative prognosis and recovery times can significantly influence athletes' and coaches' expectations, as well as medical decisions. Therefore, an in‐depth review of the existing literature is essential to provide evidence‐based guidance on returning to sports after surgical interventions such as ORIF, CRIF and AORIF.

Management of ankle injuries is not limited to surgical repair alone. Postoperative rehabilitation aspects are equally crucial to ensure a safe and effective return to sports activities. Intensive rehabilitation programs, including muscle strengthening exercises, proprioception and functional training are fundamental to restoring full joint functionality. Several studies have shown that a well‐structured rehabilitation protocol can significantly reduce recovery times and improve long‐term outcomes [19].

Bone stress injuries, for instance, are common among athletes and require particular attention in the context of rehabilitation and return to sport. Recent studies have highlighted that recovery time and return to sports activities vary significantly depending on the severity of the injury and the type of treatment administered [5]. In a sports context, the timeliness of returning to competition is often crucial but must be balanced with the need to avoid recurrence or further damage [11].

Reducing recovery times and improving functional outcomes are crucial objectives in managing ankle fractures. The introduction of less invasive techniques, such as CRIF, ARIF and AORIF, has shown promising results in reducing immobilisation times and facilitating quicker rehabilitation compared to traditional methods [29]. However, it is also necessary to consider the potential risks and complications associated with these interventions, including infections, joint stiffness and soft tissue damage [46]. Additionally, the specific fracture pattern may not always permit closed treatment, necessitating open ORIF intervention.

Another important aspect to consider is the prevention of ankle fractures. Prevention programs that include balance exercises, muscle strengthening, proprioception and flexibility have been shown to be effective in reducing the incidence of ankle trauma [28]. Awareness of the importance of prevention has grown in recent years and many sports organisations are integrating these programs into their training protocols.

This study aims to elucidate the prognosis of athletes with ankle fractures and to provide guidance on the timeline for resuming sports activities. A thorough understanding of these aspects can help optimise treatment strategies and improve long‐term outcomes for athletes. The combination of advanced surgical approaches, effective rehabilitation protocols and injury prevention programs represents the key to ensuring a safe and durable return to sports activities for athletes at all levels.

## MATERIALS AND METHODS

The review adhered to the PRISMA (Preferred Reporting Items for Systematic Reviews and Meta‐Analyses) guidelines [33], ensuring a comprehensive and systematic approach to data retrieval and synthesis.

### Search strategy

The analysis was conducted using the keywords ‘ankle fracture’, ‘tibia fracture’, ‘fibula fracture’, ‘peroneal fracture’, ‘malleolar fracture’ and ‘sport return’. Databases searched included Medline (PubMED), Cochrane and Google Scholar from 1 January 2008 to 12 April 2024. Articles published in English, Spanish, French, Portuguese and Italian in peer‐reviewed journals were considered. Excluded were biomechanical reports, animal studies, cadaver studies, in vitro research, case reports, case series with fewer than 10 cases, literature reviews, technical notes, letters to editors and instructional materials. Two authors independently reviewed abstracts and full texts were obtained if abstracts were inconclusive. All differences between the reviewers were discussed and if disagreement remained the senior author was consulted. Reference lists of selected articles were manually checked. All the selected studies were retrospectively analysed by an author who then extracted and entered the data in an Excel worksheet. Lastly, the data sheet was reviewed by two authors who agreed on the extracted data. The literature references of identified papers were also searched to find further relevant articles. All journals were considered.

### Inclusion and exclusion criteria

The eligibility criteria for inclusion in our analysis were set to ensure the selection of studies meeting rigorous standards. Studies were included if designed as an RCT or case series with more than 10 patients. Exclusion criteria were studies designed as systematic review, meta‐analysis, experimental studies (in vitro studies, animal studies or cadaveric studies), other inflammatory focus (tendinitis, etc.). The inclusion criteria for the selected articles include: acute or stress ankle fractures (talus included), elite or recreational or competitive athletes, time taken to return to activity, sport or competition; while the exclusion criteria include exercisers population, paediatric fractures, soft tissue injuries to the ankle, osteochondral lesions, arthroscopic treatment (Table 1). We considered all athletes, regardless of whether they were elite, competitive, or recreational, as many studies do not specify the competition level and the training volume in their analyses. However, we excluded exercisers from the study, focusing solely on individuals who engage in physical activity with the intent of improving performance in a competitive or systematic context. Additionally, although Paralympians meet the criteria through rigorous training and competitive intent, none of the studies included in our review addressed Paralympic athletes. Therefore, this category is not represented in our analysis.

**Table 1:** Inclusion and exclusion criteria.

	**Inclusion**	**Exclusion**
Population	Athletes with closed ankle fractures (talus included)	Exercisers population, Paediatric population, individuals with non‐sports injury, open fracture, others fractures associated, soft tissue injuries, osteochondral fractures
Intervention	ORIF, CRIF, AORIF, MIPO with or without ligament and syndesmosis repair associated	Conservative treatment, ARIF
Design	Randomised controlled trials, clinic trials and controlled trials	Other designs (e.g., systematic review, opinions commentaries and case report)
Other	English, Spanish, French, Portuguese and Italian	Not in English, Spanish, French, Portuguese and Italian

Abbreviations: ARIF, arthroscopic reduction and internal fixation; AORIF, arthroscopic‐assisted open reduction and internal fixation; CRIF, closed reduction and internal fixation; MIPO, minimally invasive plate osteosynthesis; ORIF, open reduction and internal fixation.

The three reviewers evaluated the full text of the selected articles to determine whether it was eligible for inclusion and collected data of interest. In case of doubt regarding the inclusion of an article, the senior author made the final decision. The three authors independently assessed the risk of bias. A supervisor was consulted in case of disagreement. To assess the quality of the studies, the Coleman Methodology Score (CMS) was used, which assesses methodology with 10 criteria, giving a total score between 0 and 100. A score of 100 indicates that the study largely avoids chance, various biases, and confounding factors. The subsections that make up the CMS are based on the subsections of the Consolidated Standards of Reporting Trials (CONSORT) statement (for randomised controlled trials) and are modified to allow for other trial designs. The Coleman criteria were modified to make them reproducible and relevant for this systematic review.

### Data extraction and analysis

Detailed information was systematically extracted from each selected study. The selected studies covered a range of variables including demographic data, type of fracture, surgical methods, and outcomes related to return to sports. Statistical analysis was performed using SPSS 18.0 for Windows (SPSS Inc., Chicago, IL, USA). Descriptive statistics were used to summarise the findings across all the included studies.

## RESULTS

### Search and literature selection

**Figure 1:**
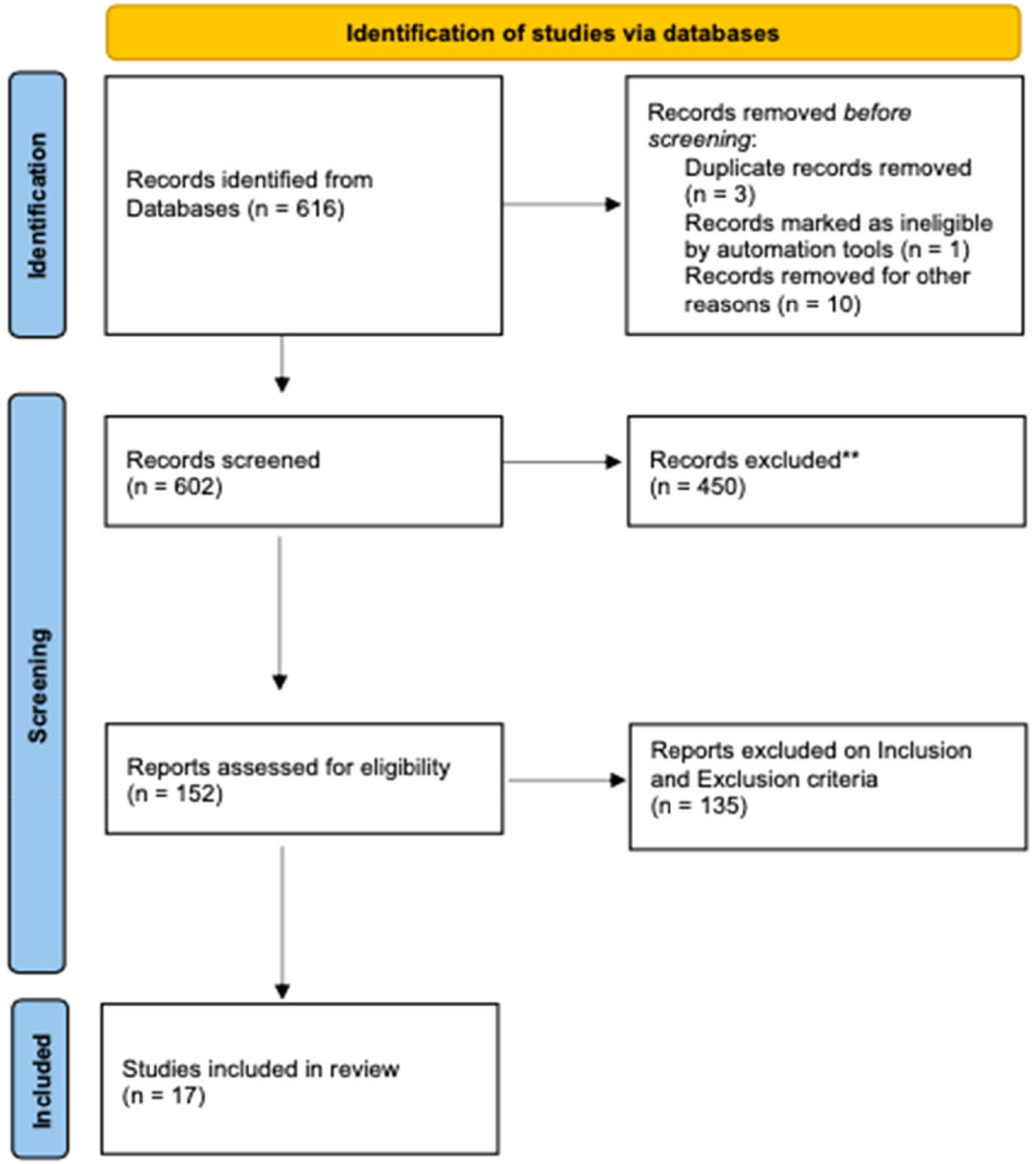
Preferred Reporting Items for Systematic Reviews and Meta‐Analyses (PRISMA) flow‐chart.

The data analysed come from scientific studies published between 2008 and 2024. An initial literature search identified 616 papers for potential evaluation. Before starting the screening process, 14 papers were excluded, leaving 602 for further review. Out of these, 450 were discarded after reviewing their titles and abstracts, as they did not meet the inclusion criteria. From the remaining studies, 135 were eliminated based on detailed inclusion and exclusion criteria. In the end, 17 papers fulfilled all the required criteria for inclusion (Figure 1).

### Demographics

This systematic review includes data from 17 studies, encompassing a total of 1422 patients who underwent surgical treatment for ankle fractures. The mean age of participants varied considerably across the studies, with an average age of 31.4 years; however, eight articles did not specify the age, although they stated that all enroled patients were adults. In terms of gender distribution, 510 patients were male (59.1%) and 354 were female (40.9%) (Table 2). Notably, some studies did not report gender distribution. Regarding sport type, 68.1% of patients were classified as general athletes without specification of the discipline practiced. Football players constituted 27.3% of the cohort, whereas young multisport athletes represented 1.6%. Concerning the level of competition, approximately 5.2% of patients were professional athletes, and 26.7% were competitive or amateur athletes. The remaining 68.1% had an undefined competitive status. It is noteworthy that most of the included studies lacked detailed reporting on both sport type and competitive level: specifically, 11 of the 17 studies (64.7%) did not provide such information, thereby limiting the ability to stratify the population based on athletic background.

**Table 2:** Demographic characteristics.

Research study	Year	No. patients	Gender	Mean age (years)	Type of sport	Competition level	CMS
Robbins et al. [38]	2024	31	31 M	25.8	Football	Professional	34
de Olivera et al. [31]	2021	49	22 M; 27 F	45	n/s	n/s	44
Lee et al. [23]	2022	100	39 M; 61 F	46.9	n/s	n/s	50
Pina et al. [34]	2021	92	64 M; 28 F	26.11	n/s	Amateur or Competitive	33
Horterer et al. [16]	2019	22	16 M; 6 F	32	n/s	n/s	40
Johnson et al. [18]	2019	43	39 M; 4 F	26	n/s	Professional	49
Robertson et al. [40]	2012	357	n/s	n/s	Football	Amateur (95%) Competitive (5%)	44
Smeeing et al. [43]	2018	115	n/s	41.5	n/s	Amateur	78
Matthews et al. [27]	2018	144	n/s	n/s	n/s	Amateur	54
Saxena and Yun [41]	2017	17	n/s	n/s	n/s	Amateur	56
Orr et al. [32]	2015	12	11 M; 1 F	29.1	n/s	Professional	28
Robertson et al. [39]	2014	96	n/s	n/s	Football (51%), Rugby (16%), Running (5%), Ice skating (3%)	Professional (5%); Amateur (40%); School level (9%); Leisure (46%)	59
Hong et al. [15]	2014	31	n/s	n/s	n/s	Amateur	41
Hong et al. [14]	2013	47	n/s	n/s	n/s	Amateur	49
Lempainen et al. [24]	2012	10	n/s	n/s	Athletics	Professional	19
Colvin et al. [6]	2009	233	269 M; 219 F	42.5	n/s	Amateur and Competitive	52
Porter et al. [35]	2008	23	19 M; 8 F	18.1	Football (45%)	Amateur and Competitive	49
Total	–	1422	510 M; 354 F	31.4	–	–	–

Abbreviations: CMS, Coleman Methodology Score; F, female; M, male; n/s, not specified.

### Type of fractures

Our review covers a diverse range of ankle fracture types, with follow‐up periods varying significantly across the studies, ranging from 8 weeks to 44 months. The average follow‐up period is 18.05 months, although Lempainen et al. and Robbins et al. didn't specify it in their study [24, 38]. The most common reported fractures include 451 unimalleolar, 153 bimalleolar and 186 trimalleolar fractures. Johnson et al. and Pina et al. focused on unstable ankle fractures [18, 34]. De Oliveira et al. examined the outcomes of surgical treatment of malleolar ankle fractures with associated distal tibiofibular syndesmosis involvement (DTFS) [31]. Porter et al. noticed 4 DTFS in their study [35]. Robbins et al. and Robertson et al. didn't outline the type of fracture [38, 40]. Maisonneuve fractures were detected in three cases [23]. One patient was diagnosed with pilon fracture [31]. The Weber classification system was frequently used: it is based on the level of the fibular fracture relevant to the tibio‐fibular syndesmosis [34]. In this literature review, we found 32 fractures classified as Weber A, 319 as Weber B and 47 as Weber C. Lempainen et al. evaluated stress fractures [24]. The Lauge‐Hansen system [22] was applied in studies focusing on the mechanism of injury: Smeeing et al. recorded supination external rotation stage 2–4 ankle fractures over a 12‐week follow‐up [43], Robertson et al. documented supination and pronation external rotation, supination adduction and pronation abduction [40]. Khare et al. used both the Lauge‐Hansen and Pott's classification [21] systems to categorise lateral and medial malleolar fractures, with a follow‐up of 36 months [40]. The Hawkins classification [1] was applied in the context of fractures of the lateral process of the talus: Horterer et al. adopted this system over a 44‐months follow‐up [16].

### Type of treatment

Seventeen studies (89.4%) are based on ORIF treatment. One study (5.2%, Saxena et al.) utilises a MIPO treatment [41] and in two articles (10.5%, De Olivera et al. and Pina et al.) deltoid ligament repair and syndesmosis repair were included in addition to ORIF [31, 34]. Horterer et al reported fractures concerning the lateral process of the talus: patients with Hawkins type I were treated with ORIF, while Hawkins type II and III were treated with open fragment removal [16] (Table 3).

**Table 3:** Type of fractures and treatment.

Research study	Number of patients	Type of fracture	Associated injuries	Main follow‐up (months)
Robbins et al. [38]	31	n/s	n/s	n/s
de Olivera et al. [31]	49	n/s	Distal tibiofibular syndesmosis	34.1
Lee et al. [23]	100	Unilteral malleolar fracture	n/s	29
Pina et al. [34]	92	Unimalleolar, Bimalleolar, Trimalleolar	Deltoid ligament rupture	12
Horterer et al. [16]	22	LPTF	n/s	44
Johnson et al. [18]	43	unstable ankle fractures	n/s	6
Robertson et al. [40]	357	Unstable ankle fractures	n/s	30
Smeeing et al. [43]	115	Lauge‐Hansen supination external rotation stage 2–4 ankle fractures	n/s	3
Matthews et al. [27]	144	Weber B	n/s	3
Saxena et al. [41]	17	Weber B	n/s	2
Orr et al. [32]	12	Unimalleolar	n/s	35.9
Robertson et al. [39]	96	Unimalleolar, Bimalleolar	n/s	36
Hong et al. [15]	31	Trimalleolar	n/s	12
Hong et al. [14]	47	Bimalleolar, Trimalleolar	n/s	12
Lempainen et al. [24]	10	Unimalleolar	n/s	n/s
Colvin et al. [6]	233	Unstable ankle fractures	n/s	12
Porter et al. [35]	23	Unimalleolar, Bimalleolar	Syndesmosis disruption, pilon fracture	28

Abbreviations: LPTF, fracture of the lateral process of the talus; n/s, not specified.

### Rate of return to sports and dropout rate

The average rate to return to sports (%RTS) is 87.1% (range 64–100). Pina et al. provides the RTS percentages for various Weber classifications: Weber A 80%, Weber B 94.4%, Weber C 91.3% [34]. Additionally, two other scientific studies (10.5%, Hong et al. and Hong et al.), addressing trimalleolar fractures with very close follow‐up (12 months), report RTS percentages of 33.3% and 27.3%, respectively [14, 15]. Three authors (21.1%, Colvin et al., Matthews et al. and Saxena et al.) don't specify the rate to return to sports [6, 27, 41]. Although most studies identified RTS as the primary outcome, the operational definition of RTS varied considerably. Some studies assessed return to participation, defined as the resumption of training or non‐competitive sporting activities, whereas others evaluated return to previous performance, conceptualised as the recovery of pre‐injury competitive levels. Horterer et al. [16] reported that although 100% of patients resumed sports participation, only 50% achieved their prior performance level. Robbins et al. [38], focusing on professional athletes, specifically examined return to performance, underscoring the distinction between participation and full functional recovery. Beyond functional outcomes, 12 of the 17 studies also reported the incidence of sports dropout following ankle fractures. The mean dropout rate across all studies was 6.8%. More specifically, dropout rates were approximately 2% among professional athletes, 5% among competitive athletes, and 8% among amateur and recreational athletes. De Oliveira et al. [31] noted that 6.1% of athletes failed to resume routine activities, whereas Pina et al. [34] documented an 8.7% dropout rate after initial RTS. Notably, Horterer et al. [16] reported no cases of sport discontinuation within their cohort. Despite the generally high rates of RTS, a substantial proportion of athletes do not regain their pre‐injury performance level, and some ultimately discontinue sporting activities following their return.

### Time to RTS

Nine authors (47.3%) don't report the time to RTS; the average time to RTS is 134.5 days (Table 4). Horterer et al. analyze the lateral process of the talus and indicate a Time to RTS of 240 days: they report that Hawkins classification has no significant influence on time to RTS [16]. Hong et al. exclusively analyze bimalleolar and trimalleolar fractures and describe a Time to RTS of 720 days [14]. The analysis demonstrated that more severe injuries, including bimalleolar and trimalleolar fractures, were associated with prolonged recovery times relative to unimalleolar fractures. Moreover, higher levels of athletic competition correlated with longer recovery periods. Colvin et al. [6] observed that only 11.6% of competitive athletes successfully returned to sport within 1 year following surgical management of ankle fractures.

**Table 4:** Rate of return to sports, time to return to sports and outcomes.

Research study	Number of patients	Type of treatment	% RTS	Time to RTS (days)	Dropout rate	Outcomes
Robbins et al. [38]	31	ORIF	71%	97.6	n/s	n/s
de Olivera et al. [31]	49	ORIF	93.9%	n/s	6.1%	98% fully satisfied, 2% partial satisfaction
Lee et al. [23]	100	ORIF	100%	n/s	n/s	FAOS
Pina et al. [34]	92	ORIF	Unimalleolar 100% Bi/trimalleolar 89%	n/s	8.7%	n/s
Horterer et al. [16]	22	ORIF	100%	240	0%	VAS FA 77 ± 21, Karlsson Score 72 ± 21
Johnson et al. [18]	43	ORIF	81%	n/s	4,6%	SANE
Robertson et al. [40]	357	ORIF	83%	182	14%	n/s
Smeeing et al. [43]	115	ORIF	n/s	n/a	n/s	OMAS
Matthews et al. [27]	144	ORIF	n/s	n/a	n/s	n/s
Saxena and Yun [41]	17	MIPO	n/s	129	0%	Roles and Maudsley score
Orr et al. [32]	12	ORIF	64%	n/s	17%	n/s
Robertson et al. [39]	96	ORIF	87%	245	6%	n/s
Hong et al. [15]	31	ORIF	33.3%	n/s	25%	OMAS, VAS
Hong et al. [14]	47	ORIF	27.3%	720	18,2%	OMAS, VAS
Lempainen et al. [24]	10	ORIF	90%	120	10%	n/s
Colvin et al. [6]	233	ORIF	n/s	n/s	n/s	n/s
Porter et al. [35]	23	ORIF	96.3%	n/s	3,7%	AAOS lower limb score module

Abbreviations: FAOS, Foot and Ankle Outcome Score; MIPO, minimally invasive plate osteosynthesis; n/s, not specified; OMAS, Olerud Molander Ankle Score; ORIF, open reduction and internal fixation; RTS, return to sport; SANE, single assessment numeric evaluation; VAS, Visual Analogue Scale.

### Clinical outcomes

Lee et al. evaluated the functional outcome using the Foot and Ankle Outcome Score (FAOS), demonstrating significant improvement over time. At 3 months, the average score for the sport section was 72.3 for immediate weight bearing (IWB) and 67.4 for delayed weight bearing (DWB). By 12 months, these scores had increased to 85.6 for IWB and 83.5 for DWB. Other FAOS subscores, including pain, symptoms, activities of daily living (ADL), and quality of life (QOL), showed similar improvements, with one‐year scores ranging from 87.3 to 90.5 for IWB and from 88.3 to 89.8 for DWB [23]. Global assessments, such as the Visual Analogue Scale (VAS), indicated a reduction in pain, with an average score of 2.05 at 12. Hong et al. detected an average Olerud‐Molander Ankle Score (OMAS) of 78.3 at 12 months, with slightly higher scores (81.7) for bimalleolar fractures compared to trimalleolar fractures (78.3) [14]. Smeeing et al. used the SF‐36 score for physical functioning that exceeded 90 for fractures treated with immediate weight bearing and showed improvements over other treatment regimens [43]. The OMAS was higher in the unprotected weight‐bearing group after 6 weeks (61.2 ± 19.0) compared to the protected weight‐bearing (51.8 ± 20.4) and unprotected non‐weight‐bearing groups (45.8 ± 22.4). Johnson et al. reported the SANE (Single Assessment Numeric Evaluation) score and revealed that 50% of patients had scores above 90, reflecting high patient satisfaction [18]. Saxena et al. adopted the Roles and Maudsley score at eight weeks with an average value of 1.0 ± 0.0 [41]. Porter et al. utilised the AAOS Lower Limb Score Module: the mean scores were 94.6 for function and 98.0 for pain at the final evaluation (12 months–3.7 years after surgery) [35] (Table 4).

## DISCUSSION

This study highlights the variability in return‐to‐sport timelines and functional outcomes post‐ankle fracture, surgically treated, providing crucial insights into the complexities and variances in recovery among athletes. Factors such as fracture complexity and rehabilitation intensity play significant roles. Our study aims to underscore the multifaceted nature of treatment outcomes, influenced by factors such as the fracture type, treatment modality and individual patient characteristics. Malleolar ankle fractures are frequent injuries [4, 36]. The first modern classification of ankle fractures, attributed to Ashurst and Bromer in 1922, was primarily based on the mechanism of injury [3]. This classification was further refined by Lauge‐Hansen in 1948 and is still in use today. The Lauge‐Hansen classification describes ankle fractures based on how the injury occurs, considering the foot's position (supination or pronation) and the force's direction (external rotation, abduction or adduction). Ligament injuries are closely linked to the fracture mechanism. Supination‐external rotation fractures often involve the anterior and posterior tibiofibular ligaments and sometimes the deltoid ligament. Supination‐adduction fractures typically affect the lateral ligament complex. Pronation‐external rotation and pronation‐abduction fractures commonly involve the deltoid ligament and syndesmotic ligaments. These patterns highlight how ligament injuries correlate with specific fracture types, guiding diagnosis and treatment [42]. While useful for describing the mechanism of injury, it is challenging to reproduce and its clinical relevance is limited by variable intra‐observer reliability [30, 37]. The Weber classification categorises ankle fractures based on the location of the fibular fracture relative to the syndesmosis. It has three types: Type A (below the syndesmosis), Type B (at the level of the syndesmosis) and Type C (above the syndesmosis). It is repeatable, comparative and easy to use [20].

In our review, fractures were managed with ORIF, particularly in cases requiring early mobilisation, as well as ARIF and AORIF. Operative management was often associated with a quicker return to sports, given its efficacy in ensuring early mobilisation and stability. Lee et al. focused on lateral malleolus fractures and found that immediate weight‐bearing resulted in a quicker return to sports, with significant improvements observed within 6–9 weeks [23]. Saxena and Yun's study on percutaneous plating for distal fibular fractures reported a 100% return rate, with patients resuming activity around 4.3 months post‐surgery [41]. Lempainen et al.'s study on medial malleolar stress fractures emphasised that early surgical intervention led to a 90% return to sports within 3–4 months, with excellent clinical outcomes [24].

However, non‐operative management has shown promising results in stable, undisplaced fractures, sometimes facilitating a faster return to activity due to the avoidance of surgical recovery time. Some studies suggest that a conservative approach in certain fracture types may be just as effective as surgical intervention, challenging the traditional preference for ORIF in all cases [17]. Anatomic reduction of the ankle joint is crucial for long‐term treatment success, regardless of the method used [3]. In the study of Porter et al., surgical reduction with rigid internal fixation was recommended to athletes with ankle fractures if any fragments of bone were displaced greater than or equal to 3 mm, or if the athlete was especially enthusiastic about a rapid return to sports [35]. Rigid anatomic internal fixation aims to stabilise the ankle for early range of motion (ROM) and weight bearing. Early ROM promotes controlled ligament stress to strengthen ligaments, reduces the risk of muscle atrophy and arthrofibrosis and facilitates a faster return to function. Early protected weight bearing improves lateral ankle ligament stability and further helps by reducing muscle atrophy, joint stiffness, and proprioception dysfunction [37]. Smeeing et al.'s research indicated that patients who engaged in unprotected weight‐bearing returned to sports earlier, typically within the first 6 weeks, with better short‐term functional outcomes [43]. Matthews et al.s' ongoing study suggested that early motion and exercise post‐surgery might reduce recovery times compared to traditional immobilisation [27].

The results reveal significant variability in RTS rates and recovery times depending on the type of surgery, fracture complexity and associated injuries. The average RTS percentage across studies is 78.6%, though this varies. Lee et al. reported a 100% RTS rate while Hong et al. observed much lower rates (33.3% and 27.3%) in more complex bi‐ and trimalleolar fractures, with recovery times extending up to 720 days [14, 23]. Robertson et al. reported an RTS rate of 87% with a mean recovery time of 245 days for unimalleolar and bimalleolar fractures [39]. Saxena and Yun, using a less invasive Minimally Invasive Plate Osteosynthesis (MIPO) technique, achieved faster recovery at 129 days, though RTS percentages were not specified [41]. This suggests that less invasive techniques might expedite recovery but the lack of comprehensive RTS data limits definitive conclusions. Fractures with associated injuries, such as syndesmosis disruption or ligament rupture, show slower recovery as well. Porter et al. reported unimalleolar and bimalleolar fractures complicated by syndesmosis disruption and pilon fractures, leading to extended follow‐up periods of 28 months [35]. Similarly, Horterer et al. reported prolonged recovery times of 240 days for LPTF [16]. These discrepancies may arise from varying definitions of RTS, population differences and injury severity. The diverse outcomes highlight the importance of considering patient‐specific factors, surgical techniques and the nature of associated injuries when evaluating recovery timelines and functional outcomes.

## STUDY LIMITATIONS

This review is subject to several limitations. First, there was considerable heterogeneity among the included studies, with wide variability in reported outcomes and a lack of standardised measures for assessing return to sport following ankle fracture surgery. The available literature remains largely non‐evidence‐based, with limited high‐quality data specifically addressing athletic populations. Furthermore, by restricting inclusion to randomised controlled trials, clinical trials, and controlled studies, we may have introduced selection bias, particularly in the form of study design bias and publication bias. RCTs and controlled trials typically apply strict inclusion and exclusion criteria, often enroling highly selected patient cohorts that may not accurately represent the broader, more heterogeneous population of athletes encountered in clinical practice. This limitation potentially compromises the external validity and applicability of our findings. Publication bias must also be considered, as studies reporting positive or statistically significant outcomes are more likely to be published, whereas those with negative or inconclusive results are underrepresented. This phenomenon could artificially inflate perceived success rates and shorten reported recovery timelines. Taken together, these limitations underscore the need for cautious interpretation of the current evidence and highlight the importance of future research incorporating more pragmatic study designs, real‐world athlete populations, and standardised outcome reporting to provide more comprehensive and generalisable insights.

## CONCLUSION

Effective surgical management of ankle fractures in athletes, including ORIF, generally leads to high rates of return to sports. However, the time to return and the functional outcomes can vary significantly based on the type and severity of the fracture, as well as the surgical and rehabilitation approaches used. The current body of literature highlights both the progress made and substantial gaps that remain. While there is a valuable information on the efficacy of various treatment modalities, the inconsistency in reporting and the methodological limitations of existing studies necessitate more rigorous research. Standardised, high‐quality studies are needed to provide clearer guidelines and improve the evidence base for returning to sports post‐injury.

## AUTHOR CONTRIBUTIONS

*Conceptualisation*: Giovan Giuseppe Mazzella, Andrea De Fazio, and Raffaele Vitiello. *Methodology*: Giovan Giuseppe Mazzella and Raffaele Vitiello. *Data curation*: Giovan Giuseppe Mazzella, Guido Bocchino, and Giacomo Capece. *Formal analysis*: Giovan Giuseppe Mazzella, and Raffaele Vitiello. *Investigation*: Giovan Giuseppe Mazzella, Guido Bocchino, Giacomo Capece, and Andrea De Fazio. *Validation*: Giovan Giuseppe Mazzella, FF, GM, Raffaele Vitiello. *Writing–original draft preparation*: Giovan Giuseppe Mazzella, Guido Bocchino, Giacomo Capece, and Andrea De Fazio. *Writing–review and editing*: Giovan Giuseppe Mazzella and Fabrizio Forconi. *Visualisation*: Fabrizio Forconi, Giulio Maccauro and Raffaele Vitiello. *Supervision*: Raffaele Vitiello. All authors have read and agreed to the published version of the manuscript.

## CONFLICT OF INTEREST STATEMENT

The authors declare no conflicts of interest.

## ETHICS STATEMENT

Please include the name of the institutional review board (IRB) and the approval number. If not applicable, please state so.

## Data Availability

The data that support the findings of this study are available from the corresponding author upon reasonable request.
